# Use of Osmotic Dehydration Assisted by Ultrasound to Obtain Dried Mango Slices Enriched With Isomaltulose

**DOI:** 10.1111/1750-3841.70223

**Published:** 2025-04-26

**Authors:** Juliana Rodrigues do Carmo, Jefferson Luiz Gomes Corrêa, Amanda Aparecida de Lima de Santos, Cristiane Nunes da Silva, Cassiano Rodrigues de Oliveira, Adriano Lucena de Araújo, Rosinelson da Silva Pena

**Affiliations:** ^1^ Department of Food Science (DCA) Federal University of Lavras Lavras Brazil; ^2^ Department of Nutrition and Health (DNU) Federal University of Lavras Lavras Brazil; ^3^ Institute of Exact and Technological Sciences Federal University of Viçosa Rio Paranaíba Brazil; ^4^ Graduate Program in Food Science and Technology Federal University of Pará Belém Brazil

**Keywords:** Antioxidant activity, Bioactive compounds, Mass transfer, Palatinose, Sorption isotherms

## Abstract

Osmotic dehydration (OD) process, as a pretreatment for drying, can be used to enrich mangoes with a solute of interest and improve the nutritional and sensory values of this dried fruit. The research aimed to obtain dried mangoes enriched with isomaltulose. The incorporation of isomaltulose in mango (*Tommy Atkins*) slices was performed by ultrasound‐assisted osmotic dehydration (UAOD). Then, the treated mango was convectively dried (60°C and 1.5 m/s). The incorporation of isomaltulose at 20 min was maximum (≈ 5% solids gain) and did not differ from experiments with the longer time. Firmness, color, ascorbic acid, total phenolics, and antioxidant activity did not differ between the mangoes subjected to UAOD and the fresh ones. After drying, the treated samples presented lower water activity, higher firmness, volumetric shrinkage, and total color difference. Similar bioactive compound content was found among treated and untreated dried samples except for the carotenoids, which were lower in the treated samples. Thin‐layer drying kinetics models demonstrated excellent fits to the experimental data (*R* ^2^ ≥ 0.984, RMSE ≤ 0.0399, and *χ* ^2^ ≤ 1.7 × 10^−3^), and the Page model, considered simple and widely used for the drying kinetics of fruits, was used to construct the curves. The sorption isotherms behavior evidenced that the incorporation of isomaltulose by ultrasound resulted in a less hygroscopic product.

**Practical Application**: This research has potential applications in the food industry, particularly in creating healthier mango snacks with a reduced glycemic index by incorporating isomaltulose. The process also helps retain essential bioactive compounds and enhances product stability during storage, making it an appealing option for consumers looking for nutritious choices and for producers aiming to maintain the quality of dried foods.

## 1 Introduction

Mango (*Mangifera indica* L.) is one of the most consumed tropical fruits whose production increases yearly (Yao et al. 2020). According to Kamchansuppasin et al. (2021), the glycemic index of mango varies depending on its ripeness, from 28.1 ± 4.8 to 63.5 ± 7.1 for green and ripe mangoes, respectively. It presents high content of vitamins (A, C, E, K, B1, B2, B3, B5, B6, B12), minerals (calcium, iron, phosphorus, potassium, magnesium, zinc, manganese), dietary fibers (cellulose, hemicellulose, lignin), and antioxidants (vitamin C, ß‐carotene, dehydroascorbic acid; Jiménez‐Hernández et al. 2017). Despite this, its high moisture content makes it a highly perishable fruit, with up to 50% wasted during the postharvest period, storage, transport, and ripening (Maldonado‐Celis et al., 2019).

Processed mango is a substantial worldwide trade, particularly dried mango (Akoy 2014; Dereje and Abera 2020). Conventional drying technologies usually degrade compounds and affect food quality, prompting the food industry to seek alternatives. Osmotic dehydration (OD) can mitigate these issues and reduce energy costs (Asghari et al. 2024; Corrêa et al. 2017). OD is a process of partial water removal, often used as a preliminary step before heated air drying, aiming to reduce processing time and enhance the sensory properties of the product (Corrêa et al. 2017; Medeiros et al. 2022; Memis et al. 2024). This process involves immersing the food in a concentrated solution, promoting the partial removal of water while the incorporation of solids from the solution to the surface and interior of the biological material occurs.

Additionally, it can modify the plant tissue and alter the nutritional composition, texture, and sensory characteristics of the fruit. OD is known for its simplicity, low cost, and low energy consumption (Medeiros et al. 2019). OD has been widely combined with other treatments to enhance the achieved results. For instance, the association of OD with ultrasound stands out (Corrêa et al. 2017; Fernandes et al. 2019).

The effectiveness of ultrasound technology in food is primarily related to the strength of cavitation. This force is described as the formation, growth, and collapse of bubbles in the liquid medium, produced by pressure fluctuations generated by the passage of acoustic waves. The implosion of these microbubbles results in various mechanical effects, such as cell rupture, which contributes to the increase in mass transfer (Asghari et al. 2024; Medeiros et al. 2019; Medeiros et al. 2022; Memis et al. 2024). Thus, during OD, ultrasound can enhance mass transfer. Ultrasonic waves and osmotic pressure promote greater deformation of the tissue structure, creating microscopic channels. These channels reduce the thickness of the diffusion boundary layer and enhance the convective mass transfer in the sample (Asghari et al. 2024; Corrêa et al. 2017; Fernandes et al. 2016; Fernandes et al. 2019; Llavata et al. 2024; Memis et al. 2024). Moreover, studies demonstrated that combining osmotic dehydration with ultrasound (UAOD) resulted in greater water loss (WL) and higher bioactive compound yields than conventional OD (Memis et al. 2024).

Therefore, the OD process, with subsequent drying, could enrich this fruit with a solute of interest besides improving its nutritive and sensorial values (Abrahão and Corrêa 2023). Hypertonic sucrose solutions are commonly used in OD of fruits (Fernandes et al. 2019; Jiménez‐Hernández et al. 2017; Zhao et al. 2018), but sucrose is highly cariogenic and causes a high glycemic and insulinemic response (Abrahão and Corrêa 2023). To mitigate these drawbacks, researchers have replaced sucrose with alternative carbohydrates such as xylitol, maltitol, erythritol, isomalt, sorbitol (Mendonça et al. 2017), inulin and oligofructose (Cichowska et al. 2018), brown sugar, honey, coconut sugar, stevia, and beet molasses (Kaur et al. 2022), aiming to improve the healthfulness of the dehydrated products. In this context, isomaltulose, commercially known as Palatinose, appears as an excellent osmotic solute alternative. It is a noncariogenic carbohydrate with low glycemic and insulinemic indexes. However, due to its low solubility and high cost, the use of isomaltulose may be limited in high‐concentration formulations and large‐scale applications. The study of dried food osmotically pretreated with isomaltulose is still underexplored (Carmo et al. 2022; Lopez et al. 2020; Macedo et al. 2021).

Studies about UAOD of mango with isomaltulose, further dried or not, are not found in the literature. The present study aimed to evaluate the hygroscopic, firmness, volumetric contraction, color, ascorbic acid, carotenoids, total phenolics, and antioxidant activity of dried mango previously treated with isomaltulose UAOD. This research contributes to developing healthier mango‐based snacks with reduced glycemic index, enhanced bioactive compound retention, and improved storage stability, offering benefits for health‐conscious consumers and the food industry.

## 2 Material and Methods

### 2.1 Raw Material

Mango fruits, *Tommy Atkins* cultivar, in a half‐ripe degree of maturation, were acquired in the local market (Lavras, MG State, Brazil). The characteristics of the fruits were reddish‐green skin peel color; 84.53 ± 1.83% w.b. moisture; 12.33 ± 0.60 °B total soluble solids; and 31.13 ± 3.27 N firmness. The fruits were washed with a disinfectant solution (chlorinated water at 200 ppm) for 5 min, and then the seed and peel were removed. Slices were obtained with a stainless‐steel mold in the following dimensions: 4.00 ± 0.01 cm in length, 2.00 ± 0.01 cm in width, and 0.40 ± 0.01 cm in thickness.

### 2.2 Osmotic Solution

The osmotic solution with a concentration of 35% (w/w) was prepared with distilled water and isomaltulose (Beneo, Mannheim, Germany). The solution presented the following parameters: water activity (*a*_w_) of 0.972 (± 0.001); solubility of 0.3255 kg of isomaltulose/kg of solution; density of 1126.1 kg/m^3^; specific heat of 3.426 kJ/kg K; thermal conductivity of 0.485 W/m K; and viscosity of 3.154 mPa s at the working temperature (28°C; Carmo et al. 2022).

### 2.3 Ultrasound‐Assisted Osmotic Dehydration

For the ultrasound‐assisted osmotic dehydration (UAOD) tests, the mango slices were immersed in the aqueous solution of isomaltulose at a ratio of 1:10 (w/w) and submitted to ultrasonic waves for 10, 20, 25, 30, 35, and 40 min in an ultrasonic bath (Unique, USC‐2850, Indaiatuba, Brazil) maintained at 28°C. The bath presented a volume of 0.0095 m^3^, a frequency of 25 kHz, and an effective ultrasonic power density of 23.2 kW/m^3^.

The parameters of WL, solid gain (SG), and weight reduction (WR) were determined according to Equations (1)–(3) (Lopes et al. 2024), respectively. The moisture content of the fresh and osmotically treated samples was determined according to the AOAC (2019). The tests were conducted in quintuplicate.

(1) $$\mathrm{WL}\left(\%\right)=\frac{M_{0}x_{0}-M_{t}x_{t}}{x_{0}}\times 100$$

(2) $$\mathrm{SG}\left(\%\right)=\frac{x_{t}S_{t}-x_{0}S_{0}}{x_{0}}\times 100=\frac{x_{t}\left(1-M_{t}\right)-x_{0}\left(1-M_{0}\right)}{x_{0}}\times 100$$

(3) $$\mathrm{WR}\left(\%\right)=\frac{x_{0}-x_{t}}{x_{0}}\times 100$$

where *M* is the moisture content (w.b.; kg of water/kg of fruit), *x* is the sample weight (kg), and *S* is solid content (w.b.; kg solid/kg fruit). The subindices “0” and “*t*” refer to fresh samples and samples after the osmotic treatment, respectively.

### 2.4 Drying Experiment

Fresh and osmotically treated samples were dried in a tunnel dryer (Eco Engenharia Educacional, MD018 model, Brazil) with forced heated air (1.5 m/s, 60 °C) until the samples reached a moisture content of 11.0 ± 1.0% (d.b.) A digital scale (Marte Científica, AD33000 model, São Paulo, Brazil), ± 0.01 g precision was coupled to the system to monitor the mass variation during the experiments. The drying tests were conducted in three repetitions.

#### 2.4.1 Drying Kinetic Modeling

Table 1 presents the models from the literature used to describe the drying kinetics of the fresh and osmotically treated samples. They were fitted by nonlinear regression and goodness of fit was assessed using the coefficient of determination (*R* ^2^), root mean square error (RMSE), and reduced chi‐squared (*χ* ^2^; Junqueira et al. 2021).

**TABLE 1:** Mathematical models used to adjust drying kinetics.

Model	Equation
Diffusion approximation	$MR=a\cdot e^{-k.t}+\left(1-a\right)\cdot e^{-k.b.t}$
Logarithmic	$MR=a\cdot e^{-k.t}+c$
Page	$MR={e^{-k.t}}^{n}$
Modified page	$MR={e^{-(k.t)}}^{n}$
Henderson and Pabis	$MR=a\cdot e^{-k.t}$
Newton	$MR=e^{-k.t}$
Wang and Singh	$MR=1+a\cdot +tb\cdot t^{2}$

MR is the moisture ratio (nondimensional), *t* is the process time (s), and *a*, *b*, *c*, *k*, and *n* are constant of the models.

#### 2.4.2 Prediction of Effective Diffusivity

It was assumed that moisture diffusion was the main mechanism for drying curve modeling during the downward rate period. When moisture diffusion controls the drying rate during a downward rate period, the diffusion equation, represented by Fick's second law of diffusion at a nonsteady state, can be used with Cartesian coordinates and in the nondimensional form (Equation 4; Junqueira et al. 2022).

(4) $$\frac{\partial M(t)}{\partial t}=\frac{\partial }{\partial z}\left(D_{\mathrm{eff}}\frac{\partial M(t)}{\partial z}\right)$$

where ∂M(t) is the amount of water at the time, *D*_eff_ is the effective diffusivity, and *z* is a generic directional coordinate. The solid sample is considered a 2 L‐thick plate; initial conditions include uniform moisture, *M*_(_ *_z_*_,0)_ = *M*_0_; boundary conditions include concentration symmetry, $\frac{\partial M(t)}{\partial t}{|}_{z=0}$ and the equilibrium content on the surface of the material, *M*_(_ *_L_*_,_ *_t_*_)_ = *M*_eq_.

Considering the initial and boundary conditions, Fick's unidirectional diffusion equation (Crank 1975) becomes (Equation 5):

(5) $$MR=\left(\frac{8}{\pi^{2}}\sum_{i=1}^{\infty }\frac{1}{{\left(2i+1\right)}^{2}}\exp\left(-{\left(2i+1\right)}^{2}\pi^{2}D_{eff}\frac{t}{4L^{2}}\right)\right)$$

where *D*_eff_ is the effective diffusivity, *i* is the number of terms in the series, *L* is the characteristic length (half the thickness of the sample), *t* is the time, and MR is the nondimensional water content, which is given by Equation (6) (Junqueira et al. 2022):

(6) $$\mathrm{MR}=\frac{M\left(t\right)-M_{\mathrm{eq}}}{M_{0}-M_{\mathrm{eq}}}$$

where MR is the quotient of the difference between moisture at a time *t* (*M*(*t*)) and moisture at equilibrium (*M*_eq_) and the difference between initial moisture (*M*_0_).

#### 2.4.3 Hygroscopicity Study

The hygroscopicity study determined moisture adsorption and desorption isotherms at 25°C (Carmo and Pena 2019). The isotherms were obtained in a vapor sorption analyzer (Aqualab VSA, Decagon, Puma, WA, USA) using the dynamic vapor sorption (DVS) method, which consists of monitoring the moisture and *a*_w_ values of a sample exposed to environments with different relative humidity (RH) levels. Approximately 1 g sample was weighed in a stainless‐steel capsule in the microanalytical balance of the VSA. To obtain equilibrium data, the sample was submitted to different levels of RH induced by changes in the injection of dry and saturated vapor. The data were obtained for an *a*_w_ range between 0.1 and 0.9, and the equilibrium condition was programmed for a change in mass per change in time (trigger % *dm*/*dt* value) below 0.1 for three consecutive measures.

The moisture of the monolayer for the adsorption and desorption processes was determined from the linear and angular coefficients of the straight obtained through the linear regression of the *a*_w_/(1 – *a*_w_)*M* versus *a*_w_ correlation, using the linearized form of the Brunauer–Emmett–Teller (BET; Equation 7; Brunauer et al. 1938).

(7) $$\frac{a_{w}}{\left(1-a_{w}\right)M_{\mathrm{eq}}}=\frac{1}{M_{0}C}+\frac{\left(C-1\right)}{M_{0}C}a_{w}$$

where *M*_eq_ is the equilibrium moisture content (kg H_2_O/100 kg d.b.), *a*_w_ is the water activity (dimensionless), *M*_0_ is the monolayer moisture content (kg H_2_O/100 kg d.b.), and *C* is the constant related to the heat of sorption of the first layer on primary sites.

The Guggenheim–Anderson–de Boer (GAB) mathematical model (Maroulis et al. 1988; Equation 8) was adjusted to the experimental moisture sorption data of dried mango since the GAB model has been reported to give a good fit for sorption isotherms of several materials (Fan et al. 2019). The goodness of fit was assessed using *R* ^2^, RMSE, and mean relative deviation (*P*).

(8) $$M_{\mathrm{eq}}=\frac{M_{0}CKa_{w}}{\left(1-Ka_{w}\right)\left(1-Ka_{w}+CKa_{w}\right)}$$

*K* and *C* are the model's parameters.

### 2.5 Sample Properties

Fresh and treated samples were characterized for water activity, texture, color, ascorbic acid content, total carotenoids, total phenolics compounds, and antioxidant activity. Besides, the treated samples were characterized by volumetric shrinkage.

#### 2.5.1 Water Activity (a_w_)

The *a*_w_ of the samples was determined at 25°C with a digital water activity meter (AquaLab 3TE, Decagon, USA; AOAC 2019).

#### 2.5.2 Texture

The texture of the mango samples impregnated was measured as firmness (*N*) of the product surface using a texturometer (TA‐X2T; Stable Micro Systems, Surrey, UK) at room temperature according to the methodology of Medeiros et al. (2019), with minor modifications. The penetration tests were conducted with a cylindrical probe (TA10) of 20 mm in diameter. The parameters used were pretest and post‐test speeds of 1 and 1.5 mm/s, respectively. The penetration distance was set to 2 mm, the trigger force was 5 g, and the deformation rate of 50%. All tests were conducted in quintuplicate for each sample.

#### 2.5.3 Volumetric Shrinkage

It was determined by measuring the area and thickness of the samples. The free software Image J. 1.45 s was used to measure the area by image analysis. It provides the sample area by converting the pixels in the image into real dimensions from a known scale (Nahimana et al. 2011). The thickness was observed for each sample at five different points using a digital caliper (Western, DC‐6 model, China). The dimensionless volume (*β*) was determined according to Equation (9) (Macedo et al. 2021).

(9) $$\beta =\left(1-\frac{V_{f}}{V_{0}}\right)\times 100$$

where *V*_f_ is the apparent volume after OD and drying processing (m^3^), and *V*_0_ is the initial volume (m^3^).

#### 2.5.4 Color Evaluation

The color was evaluated with tristimulus colorimetry in a digital colorimeter (Konica‐Minolta, CR 400, Tokyo, Japan). The operating conditions of the equipment were a diffuse light/viewing angle of 0° (specular component included) and a D65 light source. The lightness (*L* ^*^ = 0 black and *L* ^*^ = 100 white) and the chromaticity coordinates (−*a* ^*^ = green and +*a* ^*^ = red, −*b* ^*^ = blue and +*b* ^*^ = yellow) were used to define the chroma value (*C* ^*^; Equation 10), and the hue angle (*h*°; Equation 11; Carmo et al. 2019). Equation (12) was used to calculate the total color difference (Δ*E*) of the treated samples concerning the fresh fruit.

(10) $$C^{*}=\sqrt{{\left(a^{*}\right)}^{2}+{\left(b^{*}\right)}^{2}}$$

(11) $$h^{\circ }=\cos^{-1}\frac{a^{*}}{\sqrt{{\left(a^{*}\right)}^{2}+{\left(b^{*}\right)}^{2}}}$$

(12) $$\Delta E=\sqrt{{\left(L_{0}^{*}-L_{t}^{*}\right)}^{2}+{\left(a_{0}^{*}-a_{t}^{*}\right)}^{2}+{\left(b_{0}^{*}-b_{t}^{*}\right)}^{2}}$$

where the subindices “0” and “*t*” refer to fresh and treated samples, respectively.

#### 2.5.5 Ascorbic Acid

The ascorbic acid analysis was performed according to Barcia et al. (2010). For extract preparation, 10 ± 0.0001 g of sample was added to 30 mL of aqueous metaphosphoric acid solution (4.5%). The solution was left to stand for 1 h in amber glass and then it was transferred to a 50 mL flask and made up to volume with ultrapure water. Afterward, the sample was filtered through quantitative filter paper (Whatman, No. 42), and the supernatant was centrifuged at 4163 × *g* for 10 min (Sigma 3K30) out at room temperature. High‐quality liquid chromatography (HPLC, Shimadzu, LC‐20AT) equipped with a UV detector (Shimadzu, SPD‐20A) was used for quantification. A Phenomenex 5 µm C18 column (250 × 4.6 mm) was used for separation at 30°C. An aqueous solution of acetic acid 0.15% (v/v; 20 µL) with a flow rate of 1.0 mL/min was used as the mobile phase. Detection was performed at 254 nm.

The ascorbic acid peak was identified according to retention time compared to standard solutions. The analytical curve was obtained from the standard chromatograms that measure the ascorbic acid peak areas under the same separation conditions applied to the samples. Standard ascorbic acid concentrations ranged from 1 to 100 mg/L, and the results were expressed as µg/g of sample (d.b.). All the analyses were conducted in quintuplicate.

#### 2.5.6 Total Carotenoid Content

Carotenoids from 1 g of sample were extracted with acetone (≈ 25 mL) by maceration with Celite followed by vacuum‐filtration. The extraction was repeated until the extract became colorless. All the filtered extracts were combined and directed to a liquid–liquid partition in a separation funnel with petroleum ether/diethyl ether (1:1, v/v) and washed with distilled water. After partition, the carotenoid extract was evaporated under vacuum (*T* < 38°C) and resuspended in petroleum ether for spectrophotometric quantification at 450 nm (Matos et al. 2019). The total carotenoid content of the samples was calculated by using the specific extinction coefficient of β‐carotene in petroleum ether ($E_{1cm}^{1\%}=2592$; Rodriguez‐Amaya and Kimura 2004) and expressed as µg/g of sample (d.b.). All the analyses were conducted in triplicate.

#### 2.5.7 Total Phenolics Compounds and Antioxidant Activity

##### 2.5.7.1 Obtaining Extracts

The extracts for analyzing total phenolics compounds and antioxidant activity (2,2′‐azino‐bis (3‐ethylbenzothiazoline‐6‐sulfonic acid [ABTS], 2,2‐diphenyl‐1‐picrylhydrazyl radical‐scavenging activity [DPPH], and β‐carotene) were prepared according to the methodology described by Larrauri et al. (1997), with minor modifications. Firstly, the extracts were prepared by dissolving 5 g of sample in 20 mL of methanol 50%. The solution was homogenized using a shaker table (Shaker MOD. 109, Nova Ética) for 60 min. Then, the samples were filtered in filter paper and the obtained residue was homogenized for 60 min with acetone 70%. The supernatant from two extraction steps was mixed and made up to 50 mL with deionized water.

##### 2.5.7.2 Total Phenolics Compounds Determination

Phenolic compounds were estimated following the Folin–Ciocalteu method, as described by Waterhouse (2002). About 0.5 mL of sample extract, 2.5 mL of Folin–Ciocalteu solution (10%, v/v), and 2.0 mL of calcium carbonate solution (4%, w/v) were mixed and protected from light. The samples were read using a spectrophotometer (SP‐22, VIS 325–1000 nm, Biospectro, Taboão da Serra, SP, Brazil) at a wavelength of 750 nm. The results were expressed in mg of gallic acid equivalent per 100 mL (mg GAEs/100 d.b.).

##### 2.5.7.3 Antioxidant Activity Determination

The antioxidant activity of fresh and treated samples was determined by the DPPH, ABTS, and β‐carotene/linoleic acid methods. DPPH (2,2‐diphenyl‐1‐picrylhydrazyl) free radical scavenging ability was conducted according to Brand‐Williams et al. (1995). Initially, a 0.1 mM solution of DPPH was prepared by dissolving the compound in methanol. Subsequently, 1 mL of the prepared DPPH solution was combined with 50 µL of the sample. The resulting mixture was left to rest for 30 min at room temperature, shielded from light, to facilitate the reaction between the DPPH radical and the antioxidant. The absorbance readings in a spectrophotometer (SP‐22, VIS 325–1000 nm, Biospectro, Taboão da Serra, SP, Brazil) at 515 nm and the results were expressed in g/g DPPH d.b.

ABTS method followed the procedure developed by Re et al. (1999). The ABTS radical cation was formed by dissolving ABTS in water (7 mM) and reacting it with potassium persulfate (2.45 mM). The mixture was left in the dark at room temperature for 12–16 h to stabilize. Afterward, the solution was diluted in ethanol to reach an absorbance of 0.70 (±0.02) at 734 nm and equilibrated at 30°C. For the assay, 3 mL of the diluted ABTS solution was combined with 30 µL of the sample. Absorbance was recorded in a spectrophotometer (SP‐22, VIS 325–1000 nm, Biospectro, Taboão da Serra, SP, Brazil) at 734 nm after 6 min, and the results were expressed as µmol of TEs/g d.b.

The β‐carotene method was performed as previously described by Marco (1968) and modified by Miller (1971), with minor modifications. The solutions were prepared by mixing 5 mL of a β‐carotene and linoleic acid solution with 0.4 mL of the fruit extract or Trolox solutions at varying concentrations. The mixture was then incubated in a water bath at 40°C. Absorbance measurements in the spectrophotometer (SP‐22, VIS 325–1000 nm, Biospectro, Taboão da Serra, SP, Brazil) were performed at 2 min and 120 min at 470 nm. Results were expressed as % inhibition of β‐carotene oxidation.

### 2.6 Statistical Analysis

One‐way analysis of variance (ANOVA) was performed by Statistica v.10.0 (StatSoft, Inc., Tulsa, USA) to determine whether the averages' differences were significant. The differences were reported through Tukey's test at a 95% confidence interval. A Student's *t*‐test was used at a 95% confidence interval to evaluate the drying time and apparent diffusivity. The drying and sorption models were fitted by nonlinear regression using the same software and the Levenberg–Marquardt algorithm with a convergence criterion of 10^−6^.

## 3 Results and Discussion

The mango slices subjected to UAOD from 10 to 40 min had 9.23%–15.75% WL, 3.18%–5.02% SG, and 4.36%–11.67% WR. The highest WL were at 20 and 25 min, SG at 20–40 min, and WR up to 25 min, and under these conditions there was no statistically significant difference (*p* > 0.05) for each variable studied. Ultrasound enhances mass transfer in OD through acoustic cavitation, which generates microjets and turbulence, breaking down cellular barriers and increasing tissue permeability. Additionally, it reduces diffusion resistance, forms additional channels in the food, and minimizes boundary layers, optimizing water removal and solute uptake (Asghari et al. 2024; Medeiros et al. 2022; Memis et al. 2024). Thus, it is recommended to use a time of 20 min (Fernandes et al. 2019; Memis et al. 2024; Prithani and Dasha 2020) for the enrichment of mangoes with Palatinose since the increase in incorporation (SG) was not evident with the increase of the UAOD time. Moreover, a decrease in the WL and WR of the mango slices was observed. This may be linked to changes in the cellular wall of the fruit at high time in the ultrasound (Fernandes et al. 2019; Prithani and Dasha 2020).

The quick loss of water and the absorption of solids in the surface layers of the tissue at the beginning of the process can lead to structural changes that result in the compaction of these layers, increasing resistance to the transfer of water and solids and, consequently, leading to a gradual decrease in dehydration rates (Prithani and Dasha 2020). Therefore, in this study, the UAOD was performed for up to 20 min, and the samples were then submitted to convective drying (UAOD + D). Fresh fruit samples were also subjected to drying (fresh + D) to assess the impact of both processes.

### 3.1 Influence of UAOD Treatment and Drying on *a*_w_, Texture, and Shrinkage of Mango

**FIGURE 1:**
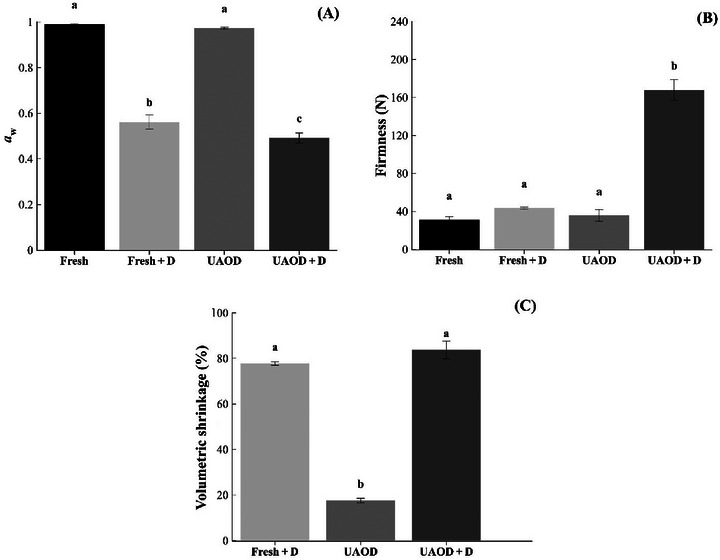
Water activity (A), firmness (B), and volumetric shrinkage (C) of mangoes subjected to the different treatment conditions; Fresh is fresh fruit, Fresh + D is dried mangoes without pretreatment, UAOD is ultrasound‐assisted osmotically dehydrated mango, and UAOD + D is dried mangoes with osmotic pretreatment assisted by ultrasound; groups with different letters differ significantly (*p* ≤ 0.05). The bar represents the standard deviation.

As shown in Figure 1A, although the UAOD did not cause a decrease in *a*_w_ compared to the fresh sample, there was a significant difference in *a*_w_ for UAOD + D in relation to fresh + D (*p* ≤ 0.05). The UAOD + D presented lower *a*_w_, as also found by Amami et al. (2017) and Medeiros et al. (2022) for strawberry and mango samples subjected to UAOD + D due to the interaction of the incorporated isomaltulose with the interior moisture (Wiktor et al. 2018). Anyway, in both dried mango slices, *a*_w_ was below 0.6, which guarantees the microbiological stability of the dried samples (De Bruijn et al. 2016).

The firmness of the samples is presented in Figure 1B. The UAOD + D sample presented higher firmness (167.95 ± 10.91 N) than fresh, fresh + D, and UAOD samples (31.13 ± 3.27, 43.65 ± 1.00, and 35.62 ± 6.16 N; *p* ≤ 0.05). This might be related to the osmotic solutes incorporated in the mango slices (Medeiros et al. 2019). Besides, according to Moreno et al. (2013) and Abrahão and Corrêa (2023), when the vegetable tissue is subjected to moisture removal, textural changes occur due to the degradation of the middle lamella, which causes loss of turgor and movement of ions from the cell wall to the media. These changes create internal stress, leading to cellular disruption and plasmolysis, such as volumetric shrinkage presented in Figure 1C.

All treatments exhibited shrinkage (Figure 1C) that, even in an osmotic or drying process, is associated with moisture removal and its consequent structural changes, as Amami et al. (2017) pointed out. The UAOD treatment resulted in approximately 17.5% shrinkage compared to the fresh sample, connected to the water loss (9.94%). The drying treatments (fresh + D and UAOD + D) had a shrinkage higher than 77% without a statistical difference between them (*p* > 0.05). These results are like those of Junqueira et al. (2017) and Corrêa et al. (2021) for shrinkage on OD and drying process, respectively.

### 3.2 Influence of UAOD Treatment and Drying on the Color of Mango Slices

**FIGURE 2:**
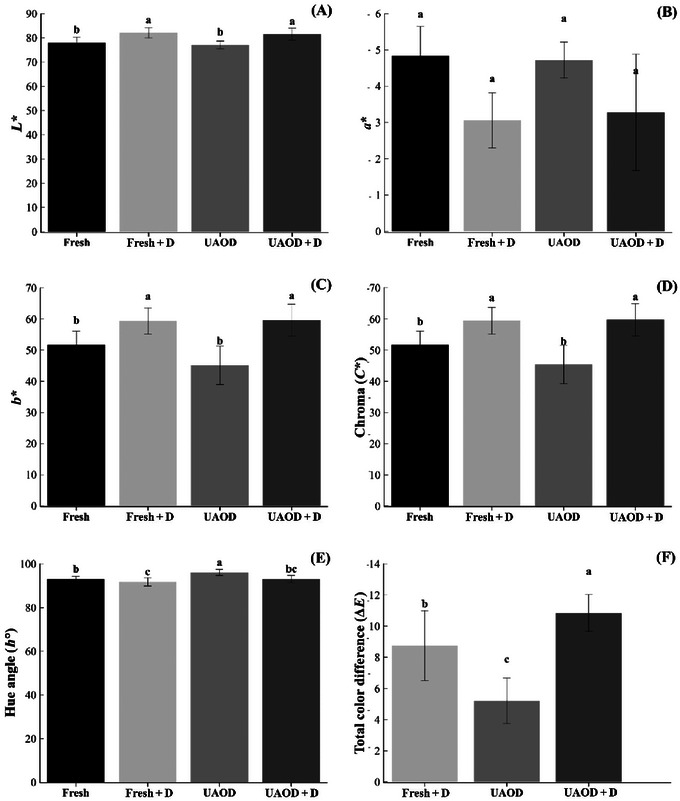
*L* ^*^ (A), *a* ^*^ (B), *b* ^*^ (C), *C* ^*^ (D), *h*° (E), and Δ*E* (F) of mangoes subjected to the different treatment conditions; Fresh is fresh fruit, Fresh + D is dried mangoes without pretreatment, UAOD is ultrasound‐assisted osmotically dehydrated mango, and UAOD + D is dried mangoes with osmotic pretreatment assisted by ultrasound; groups with different letters differ significantly (*p* ≤ 0.05). The bar represents the standard deviation.

Color is a key factor that consumers use to assess the quality of dehydrated foods, making it essential to meet their preferences (Jin et al. 2024). *L* ^*^, *a* ^*^, and *b* ^*^ values and color properties such as chroma and hue angle are used extensively to illustrate the optical attributes of fruits and vegetables (Sakooei‐Vayghan et al. 2020). According to Figure 2A isomaltulose impregnation by UAOD seemed to maintain lightness (*L* ^*^ = 77.07 ± 1.59), slight trend to green (*a* ^*^ = −4.72 ± 0.50), and yellowness (*b* ^*^ = 45.15 ± 6.16) resulting in a product close to that of the fresh fruit (*L* ^*^ = 78.88 ± 1.77, *a* ^*^ = −3.42 ± 1.43, and *b* ^*^ = 49.75 ± 4.64; *p* > 0.05). However, the dried products (UAOD + D and Fresh + D) were statistically different from the fresh and osmodehydrated samples (*p* ≤ 0.05). Generally, as is well known, the color parameters *L* ^*^ and *a* ^*^ are well correlated to color changes in fruit tissues (darkening) due to enzymatic and nonenzymatic browning reactions (Fratianni et al. 2013; Jin et al. 2024).

As browning increases, *L* ^*^ values decrease. The increase in yellowness was clear and seemed to be a result of solids uptake during osmosis pretreatment (Figure 2C). Based on the chroma value (Figure 2D), the fresh + D (*C* ^*^ = 59.41 ± 4.18) and UAOD + D (*C* ^*^ = 59.77 ± 5.14) samples presented more vivid color than the fresh sample (*C* ^*^ = 50.01 ± 4.61; *p* ≤ 0.05). The *h*° value close to 90° confirms the prevalence of the yellow color in all samples—typical of mango fruits. For both untreated and pretreated dried samples this value was lower (*h*° = 91.84 ± 1.88 and 93.12 ± 1.55) than fresh (*h*° = 93.89 ± 1.28) and UAOD (*h*° = 96.10 ± 1.37) samples (*p* ≤ 0.05; Figure 2E). These last ones had a lighter yellowish color, corroborating the trend presented by the chroma values (Carmo et al. 2019).

The UAOD in isomaltulose solutions presented a lower influence on the color changes in terms of Δ*E* in comparison to drying samples (UAOD + D and fresh + D; Figure 2F). This can be explained by the effect of the carbohydrate on reducing enzymatic browning by preventing oxygen entry (Feng et al. 2019). Further, the dried samples are more susceptible to oxidation of ascorbic acid and loss of pigments as carotenoids due to drying temperature (Fratianni et al. 2013; Santos et al. 2024).

### 3.3 Influence of UAOD Treatment and Drying on the Ascorbic Acid and Carotenoids Content of Mango Slices

**FIGURE 3:**
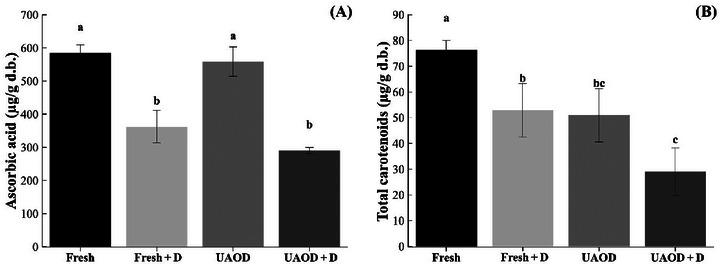
Ascorbic acid (A) and total carotenoid content (B) in mangoes subjected to the different treatment conditions; Fresh is fresh fruit, Fresh + D is dried mangoes without pretreatment, UAOD is ultrasound‐assisted osmotically dehydrated mango, and UAOD + D is dried mangoes with osmotic pretreatment assisted by ultrasound; groups with different letters differ significantly (*p* ≤ 0.05). The bar represents the standard deviation.

As presented in Figure 3A, the ascorbic acid content of UAOD samples (558.4 ± 44.2 µg/g) is not statistically different from the content of fresh samples (584.7 ± 24.2 µg/g; *p* > 0.05). As pointed out by Abrahão and Corrêa (2023), leaching of ascorbic acid from the material into the solution may occur in an osmotic process due to the high solubility of this compound in water. However, the time of the UAOD process was short. Nowacka et al. (2018) also observed that samples exposed to ultrasonic waves for up to 30 min had a slight reduction in vitamin C content. For dried samples, the ascorbic acid content was UAOD + D = 290.5 ± 9.3 µg/g and fresh + D = 362.1 ± 49.0 µg/g, exhibiting reduction (*p* ≤ 0.05), since this compound is susceptible to heat, oxygen, and light, the drying process contributes to its degradation (Medeiros et al. 2022).

A reduction in carotenoid content of UAOD + D (29.05 ± 9.28 µg/g) was observed (*p* ≤ 0.05) concerning fresh sample (76.34 ± 3.67 µg/g; reduction of 38%; Figure 3B), consistent with findings by Medeiros et al. (2022), Kroehnke et al. (2021), and Oladejo et al. (2017) for osmotically dehydrated dried mango, kiwi, and sweet potatoes. This reduction can be linked to changes in tissue structure or the leakage of these compounds into the osmotic solution (Kroehnke et al. 2021; Oladejo et al. 2017). Additionally, the effect of ultrasound energy can increase the activity of heat stable lipoxygenase. This enzyme is detrimental because it could destroy carotenoids during drying by forming reactive radicals (Cui et al. 2004). According to Medeiros et al. (2022), carotenoid degradation is influenced by both temperature and drying time. Longer drying periods intensified thermal degradation, resulting in lower carotenoid retention. This effect may explain the greater carotenoid degradation in UAOD + D treatments, where the incorporation of isomaltulose extended the drying time.

### 3.4 Influence of UAOD Treatment and Drying on Total Phenolics Compounds and Antioxidant Activity of Mango Slices

**FIGURE 4:**
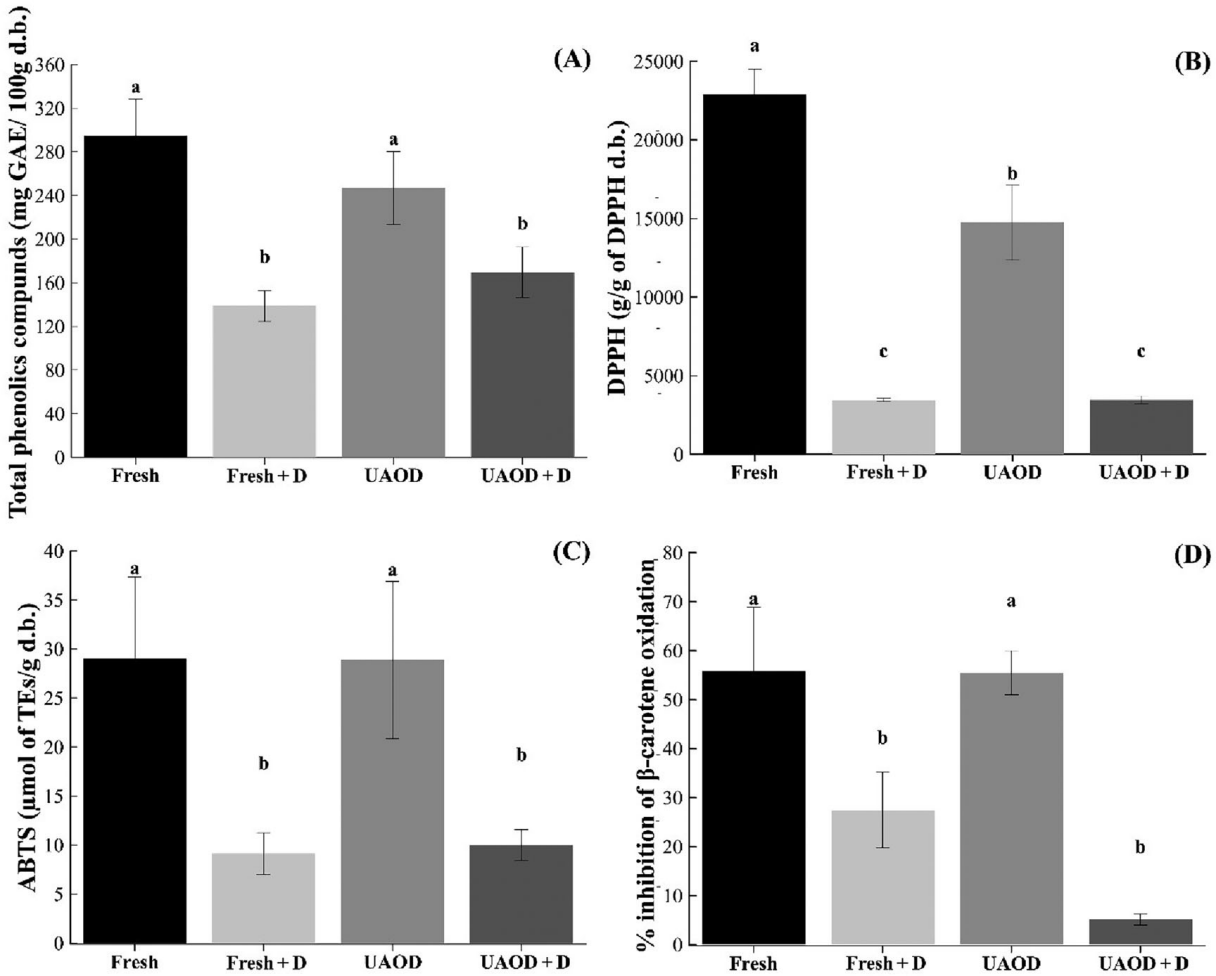
Total phenolics compounds (A), 2,2‐diphenyl‐1‐picrylhydrazyl radical‐scavenging activity (DPPH) (B), 2,2′‐azino‐bis (3‐ethylbenzothiazoline‐6‐sulfonic acid (ABTS) (C), and β‐carotene (D) in mangoes subjected to the different treatment conditions; Fresh is fresh fruit, Fresh + D is dried mangoes without pretreatment, UAOD is ultrasound‐assisted osmotically dehydrated mango, and UAOD + D is dried mangoes with osmotic pretreatment assisted by ultrasound; groups with different letters differ significantly (*p* ≤ 0.05). The bar represents the standard deviation.

Phenolics are secondary metabolites and play a significant role in food nutrition. They are nonessential compounds in dietary food (Rahaman et al. 2019). The total phenolics content of fresh mango was 294.61 (± 33.67) mg GAEs/100 g and was not significantly different from UAOD samples (246.99 ± 33.54 mg GAEs/100 g; *p* > 0.05). Reduction in total phenolics content was significantly higher in fresh + D samples (138,81 ± 14,04 mg GAEs/100 g) and UAOD + D samples (169,44 ± 23,10 mg GAEs/100 g; *p* ≤ 0.05; Figure 4A), indicating a 52.88% and 42.48% reduction in total phenolics content, respectively. This fact can be attributed to leakage to the osmotic solution, or it can be attributed to the phenomenon of acoustic cavitation induced by ultrasound and degradation during treatment at 60°C, or both. The scientific literature presents studies that reported that UAOD and drying can cause nutrient loss (Kek et al. 2013; Kroehnke et al. 2021; Mothibe et al. 2014).

As presented in Figure 4B–D, the antioxidant activity of untreated and pretreated mango samples. The fresh fruit presented 22,232.45 (± 3105.11) g/g DPPH, 29.68 (± 6.50) µmol of TEs/g, and 55.89 (±12.97) % inhibition of β‐carotene oxidation. The UAOD sample presented 14,752.73 (± 2399.72) g/g DPPH, 28.86 (± 8.03) µmol of TEs/g, and 55.51 (± 4.51) % inhibition of β‐carotene oxidation. On the other hand, the fresh + D and UAOD + D had 3451.10 (± 112.34) g/g DPPH, 9.13 (± 2.13) µmol of TEs/g, and 27.48 (± 7.70) % inhibition of β‐carotene oxidation, and 3462.80 (± 252.64) g/g DPPH, 10.00 (± 1.56) µmol of TEs/g, and 5.17 (± 1.17) % inhibition of β‐carotene oxidation, respectively.

The antioxidant activity of fresh mango comes from the presence of ascorbic acid, carotenoids, and total phenolics compounds. The analyses of DPPH, ABTS, and β‐carotene/linoleic acid method had the same tendency of these compounds: decreased with drying (*p* ≤ 0.05) and did not differ for the samples pretreated with ultrasound (*p* > 0.05) concerning fresh sample, except to DPPH. According to Amami et al. (2017), the retention of bioactive compounds depends upon osmotic solution and treatment time. Overall, the isomaltulose UAOD pretreatment of mango slices at 20 min preserved ascorbic acid, total phenolics, and their antioxidant activity.

### 3.5 Kinetics Modeling and Hygroscopicity Study

**FIGURE 5:**
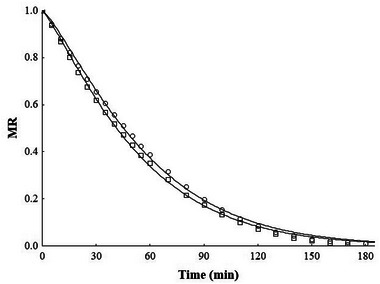
Experimental values of the evolution of the moisture ratio (MR) of dried mangoes, both without pretreatment (Fresh + D) (○) and with osmotic pretreatment assisted by ultrasound (UAOD + D) (⬜) samples overtime at 60°C. Predicted values using the Page model (line).

The tested models were able to accurately predict the drying kinetic of sliced mango since they presented *R* ^2^ ≥ 0.984 and low RMSE (≤ 0.0399) and *χ* ^2^ (≤ 1.7 × 10^−3^; Table 2). Thus, Figure 5 presents the adjustment with the Page model, which is relatively simple and was successfully used for the thin‐layer drying kinetics of several fruits and vegetables (Onwude et al. 2016).

**TABLE 2:** Mathematical modeling parameters for the drying kinetics of fresh samples (Fresh + D) and ultrasound osmotic dehydrated samples (UAOD + D) for the experimental drying data.

Model	Parameters	Samples	
		Fresh + D	UAOD + D
Diffusion approximation	*a*	−84.16	−67.12
	*k*	0.030	0.031
	*b*	0.992	0.991
	*R* ^2^	0.999	0.999
	*χ* ^2^	1.4 × 10^−4^	1.0 × 10^−4^
	RMSE	0.011	0.009
Logarithmic	*a*	1.15	1.12
	*k*	0.014	0.015
	*c*	−0.117	−0.098
	*R* ^2^	0.998	0.999
	*χ* ^2^	2.5 × 10^−4^	1.2 × 10^−4^
	RMSE	0.015	0.010
Page	*k*	0.006	0.008
	*n*	1.25	1.20
	*R* ^2^	0.999	0.999
	*χ* ^2^	1.4 × 10^−4^	1.2 × 10^−4^
	RMSE	0.011	0.010
Modified page	*k*	0.016	0.018
	*n*	1.25	1.20
	*R* ^2^	0.999	0.999
	*χ* ^2^	1.4 × 10^−4^	1.2 × 10^−4^
	RMSE	0.011	0.010
Henderson and Pabis	*a*	1.06	1.05
	*k*	0.018	0.019
	*R* ^2^	0.990	0.992
	*χ* ^2^	1.2 × 10^−3^	8.2 × 10^−4^
	RMSE	0.033	0.027
Newton	*k*	0.016	0.018
	*R* ^2^	0.984	0.989
	*χ* ^2^	1.7 × 10^−3^	1.2 × 10^−3^
	RMSE	0.040	0.033
Wang and Singh	*a*	−0.012	−0.013
	*b*	3.9 × 10^−5^	4.6 × 10^−5^
	*R* ^2^	0.999	0.997
	*χ* ^2^	8.1 × 10^−5^	2.7 × 10^−4^
	RMSE	0.009	0.016

Fresh + D is dried mangoes without pretreatment and UAOD + D is dried mango with osmotic pretreatment assisted by ultrasound; *a*, *b*, *c*, *k*, and *n* are constant of the models, *R* ^2^ is coefficient of determination, *χ* ^2^ is reduced chi‐squared, and RMSE is root mean square error.

The UAOD reduced the initial moisture content in the mango samples (78.15 ± 2.32%), and the apparent water diffusivity with pretreatment was 3.58 × 10^−10^ m^2^/s. According to Fernandes et al. (2019), the kind of tissue structure of mango slices differs from the other fruits and is less susceptible to the effects induced by ultrasound application.

The processing time required to remove 87% of the initial moisture content from mango slices was 170 ± 10 min for untreated samples and 187 ± 6 min for pretreated samples, respectively. A similar result was found by Macedo et al. (2021), with osmotically dehydrated and dried strawberry samples. This increase in drying time may be related to the absorption of solids by the sample during OD, which can cause pore clogging and hinder mass transfer. It may also be due to interactions between solute molecules and water through bond formation, making water removal more difficult and contributing to greater resistance to moisture flow during the drying process (Abrahão and Corrêa 2023; Macedo et al. 2021).

**FIGURE 6:**
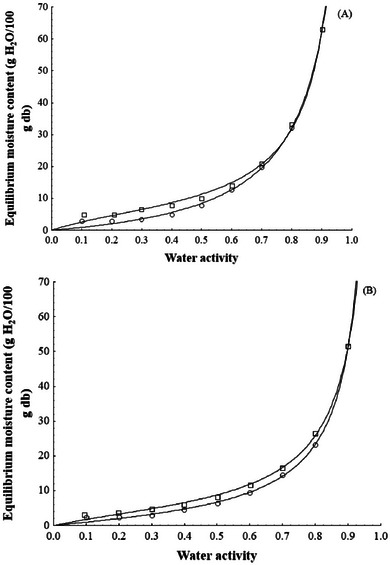
Moisture sorption isotherms of dried mangoes, both without pretreatment (Fresh + D) (A) and with osmotic pretreatment assisted by ultrasound (UAOD + D) (B). Experimental adsorption (○) and desorption (⬜) values and predicted values at 25°C using the Guggenheim–Anderson–de Boer (GAB) model (line).

Although the use of UAOD increased in the total process time, the final product incorporated 4.58 ± 0.55% isomaltulose with only 20 min of ultrasound application. Thus, this technology proves to be interesting for incorporating a carbohydrate with nutritional advantages concerning sucrose. It is worth mentioning that the mango is a fruit much less porous (porosity = 0.04–0.05) than most fruits (pineapple porosity = 0.16–0.25; apple porosity = 0.18–0.22; strawberry porosity = 0.47; Singh et al. 2015) and thus, even more promising results can be obtained for other fruits. Furthermore, the incorporation of isomaltulose resulted in a less hygroscopic product than the fresh one, as seen through the sorption isotherms (Figure 6).

The UAOD + D sample presented lower moisture content than the fresh + D for a constant *a*_w_ at 25°C, with the maximum moisture content (*a*_w_ = 0.9) 51.59 g H_2_O/100 g d.b. (Figure 6B) and 62.97 g H_2_O/100 g d.b. (Figure 6A), respectively. The lower affinity for the water molecules of the first one could be due to isomaltulose incorporation in the mango fruit. Additionally, the ultrasound produced microchannels leading to isomaltulose entry easily on mango slices (Amami et al. 2017). Noshad et al. (2012) presented that the osmosis and ultrasound pretreatment can also decrease the equilibrium moisture content of dried quince slices.

The adsorption isotherms indicate fresh + D and UAOD + D are microbiologically stable (*a*_w_ < 0.6; De Bruijn et al. 2016) when stored at 25°C if their moisture levels are 12.8% d.b. (11.3% w.b.) and 9.5% d.b. (8.7% w.b.), respectively. These results indicate that fresh + D requires greater care during storage. According to the quantitative criteria proposed by Yanniotis and Blahovec (2009) for the classification of moisture sorption isotherms, the fresh + D adsorption isotherm behaved as type III; however, the behavior of the desorption isotherm changed, and it behaved as more solution‐like type II isotherm. Similar behavior has been found by Bejar et al. (2012). On the other hand, for UAOD + D products, adsorption and desorption isotherms were classified as more solution‐like type II isotherms. This type of isotherm has also been observed for other dried fruits (Noshad et al. 2012; Sormoli and Langrish 2015).

The hysteresis loop observed between the moisture adsorption and desorption isotherms comprehended the monolayer region until approximately 0.8 and 0.9 *a*_w_ for fresh + D and UAOD + D, respectively. Hysteresis can be used as an index of the food quality. A decrease in the hysteresis loop or its complete absence has been related to greater product stability during storage (Caurie 2007). Thus, the mango dried pretreated with ultrasound can be considered a product more stable than those untreated.

The adsorption isotherms had linear behavior up to *a*_w_ of 0.5 for fresh + D and up to *a*_w_ of 0.6 for UAOD + D. After these levels of *a*_w_, the moisture content of the products increased exponentially (Figure 6). This behavior may be attributed to the dissolution of crystalline sugar at low *a*_w_ levels and the conversion of crystalline sugar into amorphous sugar at high *a*_w_ levels (Saltmarch and Labuza 1980). These results indicate that the fresh + D and UAOD + D require greater care when stored or handled in an environment with RH above 50% and above 60%, respectively. Under such conditions, the products should be stored in packaging with low water vapor permeability (Carmo et al. 2019). Another feature of the products is the presence of bioactive compounds such as ascorbic acid, carotenoids, and total phenolics (Figures 2 and 3), which are very susceptible to oxidative processes. Therefore, to minimize such processes, it is strongly indicated that the packages also have impermeability to air and do not allow light to pass through (Pombo et al. 2019).

The moisture content of the BET monolayer (*M*_0_) was 2.4 and 2.1 g H_2_O/100 g d.b. for adsorption and 4.9 and 3.4 g H_2_O/100 g d.b. for desorption in mango sample without and with pretreatment, respectively (*R* ^2^ > 0.974). The *M*_0_ values were lower in the pretreated product for adsorption and desorption, consistent with trends observed in sucrose‐treated mangoes (Falade and Aworh 2004; Zhao et al. 2018). As noted by Labuza (1984), foods with *M*_0_ ≤ 10% d.b. are considered stable; thus, both dried mangoes’ samples were considered stable products. The concept of monolayer is significant because it correlates with various aspects of physical and chemical deterioration in dehydrated foods. *M*_o_ represents the optimal moisture content that should be achieved and maintained to minimize deteriorative reactions during storage (Zhao et al. 2018).

According to the values of the statistics used to assess goodness of fit (*R* ^2^, *P*, and RMSE; Table 3), the GAB model was suitable for describing the dried mangoes' moisture adsorption and desorption processes. The isotherms generated by the GAB model are presented in Figure 6. Overall, the literature indicates the GAB model as having the best fits to the moisture sorption data of other dried products, such as apple (Prothon and Ahrne 2004), mango (Falade and Aworh 2004), mushroom (Engin 2020), fried, purple‐fleshed sweet potato slices (Fan et al. 2019).

**TABLE 3:** Parameters of the Guggenheim–Anderson–de Boer (GAB) mathematical model for the moisture sorption data of dried mangoes, both without pretreatment (Fresh + D) and with osmotic pretreatment assisted by ultrasound (UAOD + D).

Parameters	Samples			
	Fresh + D		UAOD + D	
	Adsorption	Desorption	Adsorption	Desorption
*M*_0_	10.57	7.00	5.21	5.77
*C*	0.79	4.53	1.78	3.38
*K*	0.95	0.99	1.00	0.99
*R* ^2^	0.998	0.998	0.999	0.998
*P* (%)	13.78	9.54	10.69	9.29
RMSE	0.79	1.06	0.51	0.63

Fresh + D is dried mangoes without pretreatment and UAOD + D is dried mango with osmotic pretreatment assisted by ultrasound, *M*_0_ is the monolayer moisture content (g H_2_O/100 g d.b.), *K* and *C* are the model's parameters, *R* ^2^ is the coefficient of determination, *P* is the mean relative deviation, and RMSE is the root mean square error.

## 4 Conclusions

UAOD followed by drying (UAOD + D) proved to be an effective method for incorporating isomaltulose into mango slices, enhancing both stability and nutritional value. The use of ultrasound technology is particularly promising for incorporating carbohydrates with nutritional advantages over sucrose, as it enabled the incorporation of 4.58 ± 0.55% isomaltulose in only 20 min. Ascorbic acid, total phenolics, and their antioxidant activity were preserved on mangoes subjected to UAOD treatment. The ultrasound‐pretreated dried samples presented lower water activity and similar bioactive compound content concerning untreated dried samples, except for the carotenoids, which showed a lower value. The Page model considered simple and widely used for the drying kinetics of fruits in thin layers, was used to construct the curves. Additionally, the incorporation of isomaltulose by ultrasound resulted in a more stable product, from a hygroscopic point of view, compared to the fresh‐dried one, as demonstrated by the sorption isothermal study. Despite the advantages, a reduction in carotenoid content was observed, suggesting the need for adjustments in the process to minimize losses of these compounds. However, the results indicate that UAOD + D technology is a promising alternative to produce isomaltulose‐enriched dried mango, offering a product with nutritional benefits and enhanced storage stability.

## Author Contributions

**Juliana Rodrigues do Carmo**: conceptualization, investigation, writing–original draft, writing–review and editing, validation, methodology, formal analysis, data curation. **Jefferson Luiz Gomes Corrêa**: Writing–review and editing, supervision, resources, data curation. **Amanda Aparecida de Lima Santos**: Data curation, writing–review and editing, conceptualization. **Cristiane Nunes da Silva**: Data curation, writing–original draft. **Cassiano Rodrigues de Oliveira**: Methodology. **Adriano Lucena de Araújo**: Data curation, writing–original draft. **Rosinelson da Silva Pena**: Data curation, writing–original draft.

## Conflicts of Interest

The authors declare no conflicts of interest.
